# Precision medicine in myelofibrosis: from molecular profiling to personalized therapy

**DOI:** 10.3389/fonc.2026.1877425

**Published:** 2026-07-15

**Authors:** Rafal Al-Shibly, Rasha Kaddoura, Omar Ismail, Mohamed A. Yassin

**Affiliations:** 1 Department of Medical Education, Internal Medicine Residency Program, Hamad Medical Corporation, Doha, Qatar; 2 Department of Pharmacy, National Center for Cancer Care and Research, Hamad Medical Corporation, Doha, Qatar; 3 Department of Hematology, National Center for Cancer Care and Research, Hamad Medical Corporation, Doha, Qatar; 4 College of Medicine, Qatar University, Doha, Qatar

**Keywords:** artificial intelligence, CALR, JAK2, molecular profiling, MPL, myelofibrosis, precision medicine, risk stratification

## Abstract

Precision medicine in myelofibrosis (MF) has moved beyond confirmation of a driver mutation toward an integrated interpretation of marrow morphology, molecular profile, cytogenetics, clinical phenotype, symptom burden, and transplant fitness. Myelofibrosis is biologically heterogeneous across both primary MF and secondary MF arising after polycythemia vera or essential thrombocythemia, with clinically relevant differences in disease origin, driver distribution, prognostic model validation, phenotype, and outcome. Several prognostic tools and much of the strongest molecular-risk evidence remain best validated in primary MF cohorts; therefore, PMF-derived molecular signals should be applied cautiously to secondary MF, where MYSEC-PM and disease-origin-specific interpretation are more appropriate. Current precision care is actionable now in diagnosis, integrated risk assessment, phenotype-adapted JAK inhibitor selection, structured symptom assessment, and transplant timing. Ruxolitinib remains the anchor first-line option for symptomatic proliferative disease; fedratinib provides an alternative or post-ruxolitinib option in selected patients with preserved platelet reserve; pacritinib is particularly relevant in severe thrombocytopenia; and momelotinib has a differentiated role in anemia-dominant MF. Allogeneic hematopoietic cell transplantation remains the only curative strategy and should be discussed early for biologically or clinically high-risk disease. Emerging artificial intelligence (AI) applications in digital pathology, prognostic modeling, and post-transplant prediction may further operationalize precision care, but they remain adjunctive rather than autonomous and require external validation, explainability, governance, and human oversight before routine deployment. This review reframes MF through an MF-specific precision-medicine lens centered on biology, patient selection, phenotype-adapted treatment, transplantation, monitoring, and the boundary between what is currently actionable and what remains investigational.

## 1 Introduction

Myelofibrosis is a clonal myeloid neoplasm characterized by constitutive JAK-STAT signaling, inflammatory marrow remodeling, cytopenias, extramedullary hematopoiesis, splenomegaly, constitutional symptoms, and a variable risk of leukemic transformation. Across both primary and secondary MF, modern care increasingly depends on integrated clinical, morphologic, cytogenetic, and genomic assessment rather than on a disease label alone (1–5).

This shift is clinically important because MF is not a single biologic state. Two patients carrying the same diagnostic label may have very different therapeutic priorities, including spleen and symptom control, anemia control, prevention of short-term progression, bridging to allogeneic transplantation, or referral to a clinical trial. Precision medicine in MF therefore lies not only in molecular annotation, but in translating biologic heterogeneity into more rational patient selection and timing of intervention (1, 2, 6).

## 2 Scope and aims of this review

This narrative review focuses on MF across both primary disease and secondary MF evolving from polycythemia vera or essential thrombocythemia, while acknowledging that several risk models and much of the strongest molecular literature were derived primarily from cohorts with primary MF. Because PMF and secondary MF differ in disease origin, driver distribution, validated prognostic frameworks, and some clinical trajectories, the review now explicitly separates PMF-derived molecular evidence from data that are more directly applicable to post-polycythemia vera and post-essential thrombocythemia MF. The review emphasizes MF-specific biology, diagnosis, genomic risk, patient selection, phenotype-adapted therapy, transplantation, monitoring, and emerging AI applications. The literature base was updated through April 2026, prioritizing contemporary reviews, molecular-risk studies, pivotal therapeutic trials, transplant-focused work, and translational reports with near-term clinical implications (1–4, 6, 13, 19, 32, 33).

## 3 Biological and diagnostic foundations of precision medicine in MF

### 3.1 Biology, marrow fibrosis, and disease heterogeneity

The biologic framework of MF extends beyond driver mutation status alone. JAK2, CALR, and MPL help establish clonality and activate JAK-STAT signaling, but fibrosis and clinical progression are also shaped by chronic inflammatory signaling, cytokine excess, abnormal megakaryocyte-stromal crosstalk, and remodeling of the marrow niche (2, 4, 5). This broader biology helps explain why JAK inhibition frequently improves splenomegaly and symptoms without fully eradicating the disease process.

Marrow fibrosis in MF is therefore not a purely cell-autonomous phenomenon. TGF-beta-associated signaling, inflammatory mediators such as IL-6 and TNF, and progressive disruption of the microenvironment contribute to ineffective hematopoiesis, constitutional symptoms, and stem/progenitor-cell mobilization. Precision medicine in MF must account for this interaction between clone and microenvironment, because treatment resistance and incomplete disease modification often arise from both (2, 4, 5).

### 3.2 Precision diagnosis and classification

Modern diagnosis in MF remains clinicopathologic and molecular. Bone marrow morphology is indispensable for confirming overt fibrotic disease and for distinguishing MF from other myeloid neoplasms, while driver mutation testing establishes clonality and anchors subsequent prognostic and therapeutic interpretation (1, 3).

Diagnosis should be viewed as the gateway to precision management rather than as a static label. Accurate classification determines whether broader next-generation sequencing, cytogenetic evaluation, dynamic prognostic modeling, or early transplant discussion is warranted. Triple-negative disease and atypical presentations should trigger more careful clinicopathologic review, not a premature assumption that the disease is molecularly silent (1, 3, 6).

### 3.3 Primary versus secondary myelofibrosis: implications for precision medicine

A precision-medicine framework for MF should distinguish primary MF from secondary MF arising after polycythemia vera or essential thrombocythemia. These entities share marrow fibrosis, inflammatory remodeling, splenomegaly, cytopenias, and leukemic risk, but they do not begin from the same biologic state. PMF arises *de novo* within the myeloproliferative neoplasm spectrum, whereas secondary MF reflects fibrotic evolution of an antecedent PV or ET clone. As a result, PMF-derived molecular frequencies, prognostic models, and transplant-risk literature should not be applied to post-PV or post-ET MF without acknowledging the limits of extrapolation (1–3, 33).

The practical distinction is most visible in genomic interpretation and risk assessment. In WHO-defined PMF, driver mutations are usually mutually exclusive and are best presented as PMF-specific categories, whereas post-PV MF is expected to be predominantly JAK2-mutated and post-ET MF retains a distribution closer to antecedent ET. Similarly, MIPSS70, MIPSS70+ version 2.0, and GIPSS were developed for PMF, while MYSEC-PM was designed specifically for post-PV and post-ET MF. In day-to-day care, this means that a patient with secondary MF should still undergo marrow review, cytogenetics, and next-generation sequencing, but prognostic counseling should incorporate disease origin and the model validated for that setting rather than assuming that PMF-based scores have identical performance (10–12, 33). See **Table 1**.

**Table 1:** Practical comparison of primary and secondary myelofibrosis.

Feature	Primary myelofibrosis (PMF)	Secondary MF: post-PV/post-ET	Precision-medicine implication
Disease origin	*De novo* myeloproliferative neoplasm with prefibrotic and overt fibrotic phases.	Fibrotic progression of antecedent polycythemia vera or essential thrombocythemia.	The diagnostic label carries biologic and prognostic context; prior PV/ET history should be retained in risk interpretation.
Driver mutation distribution	PMF-specific mutually exclusive distribution is approximately JAK2 58%, CALR 25%, MPL 8%, and triple-negative 9% in large annotated cohorts (32).	Post-PV MF is predominantly JAK2-mutated; post-ET MF includes JAK2, CALR, MPL, and triple-negative cases, with a distribution closer to ET/PMF than to PV (2, 33).	Driver frequencies should be stated by disease subtype; PMF percentages should not be generalized to all MF.
CALR biology	Type 1/type 1-like CALR identifies a favorable molecular signal in PMF and is embedded in MIPSS70+ v2.0 and GIPSS.	CALR-mutated post-ET MF may have more favorable behavior than JAK2-mutated or triple-negative disease, but CALR subtype effects are less consistently operationalized than in PMF.	CALR should not be collapsed into a single category when PMF prognostication is discussed.
Clinical and pathologic pattern	Often more cytopenic at presentation, with anemia and transfusion burden becoming central treatment determinants.	Often presents after a proliferative antecedent phase; post-PV MF may show more leukocytosis, splenomegaly, symptoms, and thrombotic history, while post-ET MF may retain higher platelet counts early in evolution.	Phenotype-adapted therapy should integrate disease origin, cytopenias, spleen burden, symptom score, and prior therapy exposure.
Risk tools	DIPSS/DIPSS-plus remain useful; MIPSS70, MIPSS70+ v2.0, and GIPSS incorporate PMF-specific molecular and cytogenetic variables.	MYSEC-PM was developed for post-PV and post-ET MF and includes age, hemoglobin, platelets, circulating blasts, constitutional symptoms, and CALR-unmutated genotype.	Use PMF-derived scores primarily for PMF; use MYSEC-PM or cautious integrated assessment for secondary MF.
Therapeutic strategy and outcomes	Treatment is guided by symptoms, spleen burden, cytopenias, molecular/cytogenetic risk, and transplant fitness.	Approved JAK inhibitors are used similarly, but prior PV/ET treatment history, proliferative phenotype, thrombotic burden, and MYSEC-PM risk may change timing of transplant or trial referral.	Therapy remains phenotype-led, but disease origin helps frame prognosis, monitoring intensity, and the urgency of transplant discussion.

### 3.4 Molecular architecture, clonal complexity, and genomic biomarkers

Because JAK2, CALR, and MPL driver mutations are generally mutually exclusive, their frequencies should be reported as subtype-specific categories rather than as independent percentages. In large WHO-defined PMF cohorts, JAK2 V617F accounts for approximately 58% of cases, CALR for approximately 25%, MPL for approximately 8%, and triple-negative disease for approximately 9%, thereby reconciling the major driver groups to roughly 100% (32). These figures refer to PMF and should not be assumed to describe secondary MF, in which post-PV MF is predominantly JAK2-mutated and post-ET MF shows a different driver distribution (2, 33). Driver lesions influence phenotype and help explain part of the observed heterogeneity, but they are insufficient to define prognosis or treatment urgency on their own.

Additional mutations in genes such as ASXL1, SRSF2, U2AF1, EZH2, IDH1/2, and TP53 more strongly shape adverse biology. These lesions are associated with inferior survival, cytopenic phenotypes, leukemic evolution, or reduced durability of standard therapy. Their interpretation should include both mutation identity and mutation burden. MIPSS70 and MIPSS70+ version 2.0 incorporate high-molecular-risk (HMR) mutations, and the presence of two or more HMR mutations represents a distinct adverse stratum rather than a simple yes/no finding (10, 11). U2AF1, particularly the Q157 variant, is especially relevant in anemia- and thrombocytopenia-predominant disease. TP53-mutated MF should also be interpreted by allelic state when available: single-hit TP53 does not carry the same implication as multi-hit or biallelic TP53 alteration, which is associated with aggressive biology and poor post-transplant outcomes (7, 21). See **Table 2**.

**Table 2:** Key molecular and genomic biomarkers in myelofibrosis.

Biomarker/feature	Approximate frequency or definition	Main clinical significance	Precision relevance
JAK2 V617F	PMF approximately 58%; post-PV MF predominantly JAK2-mutated; post-ET MF commonly but not universally JAK2-mutated	Canonical driver; often associated with proliferative phenotype, splenomegaly, leukocytosis, and thrombotic history	Confirms clonality, but PMF frequencies should not be generalized to secondary MF and do not by themselves select a specific approved JAK inhibitor
CALR type 1/type 1-like	CALR overall approximately 25% in PMF; type 1/type 1-like is the favorable CALR subgroup	Associated with more favorable survival in PMF and incorporated as a favorable molecular signal in MIPSS70+ v2.0 and GIPSS	Should be reported separately from type 2/type 2-like CALR when PMF prognosis is being refined
CALR type 2/type 2-like	Less common than type 1/type 1-like in PMF	Does not carry the same favorable prognostic weight as type 1/type 1-like CALR in PMF	Avoids oversimplifying CALR as one biologic category; supports more precise counseling
MPL mutation	PMF approximately 8%; also seen in post-ET MF	Driver lesion through thrombopoietin-receptor signaling	Supports diagnosis and pathway biology, but not yet drug-specific selection
Triple-negative disease	PMF approximately 9%, assay-dependent	Higher-concern molecular category with less straightforward biologic interpretation	Should trigger broader genomic work-up, careful clinicopathologic review, and closer reassessment
HMR mutations and HMR count	ASXL1, SRSF2, EZH2, IDH1/2, and U2AF1Q157; >=2 HMR mutations form a distinct adverse stratum in MIPSS70-based models	Associated with inferior survival, leukemic evolution, cytopenic phenotype, or aggressive disease behavior	Lowers threshold for transplant referral, trial prioritization, and intensified monitoring; the number of HMR mutations matters
TP53 allelic state	Single-hit versus multi-hit/biallelic TP53 alteration	Multi-hit/biallelic TP53 identifies particularly aggressive biology and poor transplant outcomes	Should be reported by allelic state when possible rather than treated as a simple positive/negative marker
Cytogenetic risk	Favorable, unfavorable, and very-high-risk categories; very-high-risk lesions include -7, inv(3)/3q21, i(17q), +21, +19, 12p-, and 11q-	Refines prognosis beyond clinical scores and mutations	Operationalizes GIPSS/MIPSS70+ v2.0; named lesions should be stated so the risk category is clinically usable

Cytogenetic information is also actionable only when risk categories are specified. In contemporary PMF frameworks, favorable karyotype generally includes normal karyotype or isolated favorable lesions such as +9, 13q-, 20q-, 1q abnormality, or -Y; very-high-risk abnormalities include -7, inv(3) or 3q21, i(17q), +21, +19, 12p-/12p11.2, and 11q-/11q23; other non-favorable abnormalities, complex non-monosomal karyotype, and monosomal patterns should be interpreted as adverse according to the model being applied (1, 11, 12). From a precision-medicine standpoint, most biomarkers in MF remain more prognostic than predictive. No baseline mutation profile yet mandates a single approved JAK inhibitor in the way some solid tumors are treated according to a dominant targetable lesion. Even so, the broader genomic and cytogenetic context already changes how intensively patients are monitored, how early transplantation is discussed, and whether standard therapy is framed as destination therapy, bridge therapy, or temporary disease control while trial referral proceeds (1, 6, 7, 10–12, 21, 32, 33).

## 4 Clinical application of precision medicine in MF

### 4.1 Integrated prognostication and treatment urgency

Risk stratification is the most mature clinical expression of precision medicine in MF. Clinical models such as DIPSS and DIPSS-Plus remain relevant because they capture disease tempo, symptomatic burden, and readily available laboratory risk variables, but integrated scores improve biologic risk discrimination by incorporating cytogenetics and adverse mutations (8, 9).

MIPSS70, MIPSS70+ version 2.0, and GIPSS reflect this evolution most clearly. These models give formal weight to adverse molecular lesions, the absence of CALR type 1/type 1-like biology, HMR mutation burden, and cytogenetic categories, thereby refining counseling, timing of transplant referral, and the threshold for intensified monitoring. Importantly, MIPSS70 and MIPSS70+ version 2.0 do not merely list HMR genes; they also recognize the adverse effect of accumulating HMR lesions, with two or more HMR mutations representing a distinct high-risk molecular state (10, 11). Most of these integrated tools were derived primarily in PMF cohorts and should be interpreted thoughtfully when extrapolated to secondary MF, where MYSEC-PM is the more directly validated disease-specific model (1, 10–12, 33).

This distinction matters in practice. A patient who appears clinically intermediate risk may be reclassified into a more urgent therapeutic category once multiple HMR mutations, multi-hit TP53 alteration, or very-high-risk cytogenetics are identified. Precision prognostication in MF therefore does not merely estimate survival; it helps define treatment urgency and shapes the balance between standard therapy, clinical trials, and transplantation (1, 6, 8–12, 21, 33).

### 4.2 Patient selection and treatment goals

Patient selection in MF remains predominantly phenotype-led rather than mutation-led, but it is increasingly sophisticated and should incorporate whether the disease is PMF or secondary MF. Before choosing therapy, the clinician should define the dominant goal of care: control of splenomegaly and constitutional symptoms, improvement in anemia or transfusion burden, management of thrombocytopenia, short-term disease control while planning transplant, or escalation because of biologically aggressive disease (13).

At minimum, patient selection should integrate complete blood counts, transfusion history, spleen burden, symptom assessment, marrow diagnosis, cytogenetics, next-generation sequencing, disease origin, performance status, and transplant fitness. Molecularly adverse disease does not preclude symptom-directed therapy, but it lowers the threshold for using standard therapy as a bridge while donor search, transplant evaluation, or clinical-trial referral proceeds. In practice, a simple algorithm is useful: proliferative symptomatic MF generally favors a ruxolitinib- or fedratinib-based pathway, severe thrombocytopenic MF favors pacritinib, anemia-dominant or transfusion-burdened MF favors momelotinib-centered care, and biologically high-risk disease should trigger earlier transplant and trial discussion rather than prolonged reliance on symptom control alone (13, 16–20). See **Tables 3**, **4**.

**Table 3:** Phenotype-adapted therapeutic selection in myelofibrosis.

Therapy/strategy	Best-fit phenotype	Main strengths	Key limitations/cautions
Ruxolitinib	Symptomatic proliferative MF with splenomegaly and adequate platelet reserve	Reference standard for spleen and symptom control	May worsen anemia or thrombocytopenia; less ideal when cytopenias dominate
Fedratinib	Need for spleen/symptom control after or instead of ruxolitinib with platelets generally preserved	Useful post-ruxolitinib option with meaningful activity	Gastrointestinal toxicity, thiamine monitoring, and platelet threshold remain important
Pacritinib	Severe thrombocytopenic or myelodepletive MF	Most phenotype-matched approved option when platelet counts are very low	Requires careful monitoring for gastrointestinal and bleeding-related toxicity
Momelotinib	Anemia-dominant MF or patients with substantial transfusion burden	Differentiated value for anemia, symptoms, and spleen burden	Not designed primarily for profound thrombocytopenia
Clinical trial/combination strategy	High-molecular-risk disease, suboptimal JAK-inhibitor response, or disease-modification goal	May move care beyond symptom control alone	Availability, eligibility, and toxicity profile vary by protocol

**Table 4:** Patient-selection framework for precision management in myelofibrosis.

Clinical scenario	Minimum baseline data	Preferred initial direction	Escalation trigger
Proliferative symptomatic MF	CBC, spleen burden, symptom score, marrow diagnosis, NGS/cytogenetics	Ruxolitinib-centered strategy; fedratinib in selected settings	Inadequate spleen/symptom response or progressive cytopenias
Severe thrombocytopenic MF	CBC trend, bleeding risk, symptom burden, organ function	Pacritinib-centered approach	Persistent clinical deterioration or inability to sustain therapy
Anemia-dominant/transfusion-dependent MF	Hemoglobin, transfusion history, symptom burden, spleen size	Momelotinib-centered strategy plus supportive care	Ongoing transfusion burden or worsening disease tempo
High-molecular-risk disease	Broad NGS, cytogenetics, transplant assessment	Early transplant referral and clinical-trial consideration	Rising blasts, worsening counts, or short-lived benefit from standard therapy
Potential transplant candidate	Performance status, comorbidity burden, donor search, molecular risk	Integrated transplant planning with symptom control as bridge therapy	Delay in donor search or narrowing fitness window
Post-JAK inhibitor suboptimal response	Prior exposure, reason for failure, current counts, spleen and symptom trajectory	Phenotype-matched switch plus trial review	Lack of clinically meaningful benefit after switching or rapid progression

### 4.3 Phenotype-adapted therapeutics in myelofibrosis

Ruxolitinib remains the anchor first-line option for symptomatic proliferative MF with splenomegaly and adequate platelet reserve. The COMFORT program established durable benefits in spleen response, symptom control, and overall disease burden, making ruxolitinib the benchmark against which other JAK-inhibitor strategies are interpreted (13–15).

Fedratinib is best considered when meaningful spleen and symptom control are needed after or instead of ruxolitinib in patients with preserved platelet counts. Updated JAKARTA2 analyses confirmed clinically relevant activity in previously ruxolitinib-treated disease, supporting its role as an important second-line or alternative option in selected patients (13, 16).

Pacritinib and momelotinib illustrate the practical strength of phenotype-matched care. Pacritinib is particularly relevant in severe thrombocytopenia, a setting in which dose-intensive use of other JAK inhibitors is often difficult, whereas momelotinib has differentiated value in anemia-dominant MF and in patients for whom transfusion burden is a major treatment priority (13, 17, 18).

The field is also moving toward combination and potentially more disease-modifying strategies. Pelabresib plus ruxolitinib has shown phase 3 activity in JAK inhibitor-naive MF and reflects a broader shift from single-pathway symptom control toward biologically rational combinations. Even so, such strategies still need to be interpreted through the lens of patient selection, tolerability, cytopenias, and transplant intent (13, 19).

### 4.4 Allogeneic hematopoietic cell transplantation, bridge strategy, and timing of referral

Allogeneic hematopoietic cell transplantation remains the only curative treatment for MF and should be integrated early for patients with biologically or clinically high-risk disease. Updated EBMT/ELN recommendations support transplant consideration not only for high clinical-risk MF, but also for selected patients whose molecular profile, disease tempo, or treatment failure suggests that delay may narrow the window of transplant eligibility (20, 21).

This is especially important in patients with adverse co-mutations, increasing blasts, refractory splenomegaly, worsening cytopenias, or poor milestone responses to JAK inhibition. In such settings, the central question is not simply whether symptoms can be palliated, but whether symptom-directed therapy is delaying definitive management. Biologically aggressive MF should therefore prompt earlier referral, with JAK inhibition often used as bridge therapy rather than destination therapy (13, 20, 21).

### 4.5 Molecular monitoring and response assessment

Molecular monitoring in chronic-phase MF remains an evolving area. Serial assessment of driver mutation burden may be informative in selected contexts, but universally accepted thresholds for routine treatment escalation or de-escalation are still lacking. At present, molecular changes should be interpreted alongside blood counts, symptoms, spleen measurements, and disease trajectory rather than in isolation (1, 13).

The post-transplant setting is more actionable. Clearance of driver mutations after allogeneic transplantation has been associated with lower relapse risk and better disease-free survival, making molecular monitoring substantially more useful after transplant than in routine chronic-phase care (22).

## 5 Emerging tools and implementation

### 5.1 Artificial intelligence and decision support in myelofibrosis

AI is no longer a purely speculative theme in MF. One of the most mature near-term use cases is digital bone marrow pathology, where quantitative image analysis has shown promise for supporting fibrosis grading and subtype recognition in myeloproliferative neoplasms. This is particularly relevant in MF, where marrow interpretation remains central to diagnosis yet vulnerable to interobserver variability (23).

A second area of promise is prognostic modeling. Machine-learning approaches such as AIPSS-MF and post-transplant prediction models suggest that individualized risk estimation may eventually become more dynamic and data-rich than current static scores. In addition, multimodal deep-learning pathology models indicate that image-based support tools may improve diagnostic standardization when integrated with clinical data (24–26).

A practical future use case is an AI-assisted decision-support layer that combines marrow pathology, blood count trends, symptom scores, transfusion history, spleen burden, cytogenetics, and genomic data to calculate risk, flag high-risk disease, and support trial or transplant referral. However, responsible implementation requires external validation, explainability, auditability, lifecycle monitoring, and regulatory oversight. Current FDA guidance on AI-enabled medical software and the evolving European AI framework make it clear that technical performance alone is insufficient for clinical deployment, and that AI in MF should function as adjunctive rather than autonomous support (26–28).

### 5.2 Patient-reported outcomes and real-world implementation

Precision medicine in MF should not be reduced to genomics alone. Symptom burden is a central part of disease biology and treatment benefit, and validated patient-reported outcome tools such as the MPN-SAF and MPN-SAF Total Symptom Score remain essential for longitudinal assessment. A patient with modest splenomegaly but severe fatigue, night sweats, pruritus, or early satiety is not clinically equivalent to a patient with the same molecular profile and minimal symptoms (29, 30).

Implementation in routine practice remains challenging. Access to extended sequencing, consistent interpretation of molecular findings, standardized symptom assessment, and multidisciplinary transplant evaluation is still variable across institutions. The most useful precision framework is therefore the one that integrates molecular data, marrow interpretation, clinical phenotype, and patient-reported burden into a single management strategy (1, 13, 31).

### 5.3 What is actionable now and what remains investigational

At present, the most actionable elements of precision medicine in MF are integrated marrow and molecular diagnosis, cytogenetic and next-generation sequencing-based risk refinement, phenotype-adapted JAK inhibitor selection, structured symptom assessment, and early transplant referral for clinically or biologically high-risk disease. By contrast, several important areas remain investigational: using baseline mutation profiles to predict the optimal approved JAK inhibitor, applying chronic-phase molecular response thresholds to routine treatment escalation, deploying AI systems as independent clinical decision-makers, and adopting combination or putatively disease-modifying strategies outside the clinical-trial setting. This distinction is important because the value of precision medicine in MF should be judged by whether it changes a real management decision today, rather than by the amount of biologic detail it can generate (1, 13, 19, 22–31).

## 6 Future directions

The next phase of progress in MF will depend on better predictive biomarkers, more standardized use of genomic data, earlier identification of biologically aggressive disease, and more deliberate integration of clinical-trial strategies into routine care. Combination therapy and biologically matched approaches may further shift practice away from symptom control alone, but their value will still depend on whether they improve meaningful clinical outcomes in clearly defined patient subsets (6, 13, 19).

Equally important is the refinement of selection frameworks that help clinicians decide when a JAK inhibitor is sufficient, when it should be used as bridge therapy, and when transplantation or trial referral should take priority. Precision medicine in MF is most valuable when it changes decisions, not merely when it adds molecular detail (1, 20).

## 7 Limitations of the current precision framework

Despite major progress, precision medicine in MF remains more prognostic than predictive. Many biomarkers refine risk without yet selecting a single preferred approved therapy, molecular monitoring in chronic-phase disease is not fully standardized, and several AI applications remain early translational tools rather than routine clinical instruments. In addition, evidence supporting integrated prognostic models and some molecularly informed strategies is stronger in primary MF than in secondary MF. PMF-specific driver frequencies, CALR type 1/type 1-like prognostic benefit, HMR-count logic, and GIPSS/MIPSS70+ v2.0 cytogenetic categories should therefore be applied with clear awareness of disease origin, and MYSEC-PM should be considered when counseling patients with post-PV or post-ET MF (1, 6, 10–12, 20, 22–28, 31–33).

## 8 Conclusion

Precision medicine in myelofibrosis is already operational in diagnosis, prognostication, phenotype-adapted therapy, transplant timing, and response assessment. Its current form is best described as integrated and biology-informed rather than fully genotype-matched, but that distinction should not obscure how substantially modern MF management has changed across both primary MF and secondary MF in day-to-day practice (1, 13, 19).

The strongest practical advances are the integration of molecular-risk data into treatment urgency, the matching of approved JAK inhibitors to platelet and anemia phenotypes, the more explicit separation of PMF from secondary MF in prognostic interpretation, the earlier use of transplantation in high-risk disease, and the emergence of AI-supported tools that may help standardize diagnosis and risk prediction. Future progress will depend on linking these advances to reproducible, patient-centered decisions while keeping clear boundaries between what is actionable now and what still belongs within validation studies or clinical trials (6, 22–33).
